# A Prospective Study Comparing the Efficacy of Local Injection of Platelet-Rich Plasma (PRP) vs Methylprednisolone in Plantar Fasciitis

**DOI:** 10.7759/cureus.25523

**Published:** 2022-05-31

**Authors:** Kishore Vellingiri, Nagakumar J S, Manohar P V, Joe P Lourdu, Meenakshi S Andra Suryanarayana

**Affiliations:** 1 Department of Orthopaedics, Sri Devaraj Urs Academy of Higher Education and Research, Kolar, IND; 2 Department of Orthopaedics, Sri Devaraj Urs Medical College, Kolar, IND; 3 Department of Biochemistry, PSG Institute of Medical Sciences and Research, Coimbatore, IND

**Keywords:** plantar fasciitis, prospective study, local injection, methyl prednisolone, platelet-rich plasma

## Abstract

Introduction

Plantar fasciitis is a common musculoskeletal problem in Orthopaedic practice. Heel pain caused due to plantar fasciitis, if persistent, can cause distress to the patient, so the correct intervention at the right time is needed. Plantar fasciitis is also common in the rural population.

Objectives

To compare the efficacy of local injection of platelet-rich plasma (PRP) and corticosteroid (CS) (methylprednisolone) in patients with chronic plantar fasciitis, and to evaluate the safety, side effect and complications of two different modalities of treatment.

Materials and methods

The study period was between August 2018 and September 2020. After obtaining proper written consent, 110 patients, who were above the age of 18 years and suffering from plantar fasciitis for more than three months, were included in the study. The patient characteristics including gender, age, weight, history of heel pain, duration of symptoms and types of prior treatment were noted. All the 110 patients were subjected to four parameter assessments before administration of the PRP/CS injections. Out of the 110 patients, 55 patients received PRP injection and 55 received CS - 2 ml (40 mg) methylprednisolone with 2 ml of sterile water injections. Post administration of injections, the patients' clinical, radiological, subjective and functional outcomes were assessed at the first, third and sixth month by using the Visual Analog Scale (VAS), Foot and Ankle Outcome Instrument Core Scale (FAI), Roles and Maudsley Scores (RMS), American Orthopaedic Foot and Ankle Society (AOFAS) ankle-hind foot scale and ultrasonogram of plantar fascia thickness.

Discussion

In this study, 110 patients were screened and evaluated. Out of these 110 patients, five patients who received PRP and five who received CS were lost for follow-up. Out of the 110 patients, 59 were females and 41 were males. The majority of the patients were in the BMI range of 18.5 to 24.9, with a mean BMI of 23.6. Comparing the results in both the groups reflected an improvement in the group of patients who received PRP injections. Two patients had post-operative complications (superficial infection) in the PRP injection group, while 10 patients had post-procedure complications (five patients developed superficial infections, three patients developed skin depigmentation, and two patients had atrophy of fat pad) in the corticosteroid injections (CSI) group. Infections subsided in all the patients as observed during subsequent follow-up.

Conclusion

This study shows that PRP administration is a good method of managing patients suffering from chronic plantar fasciitis, presenting with some discomfort following activity, with more than three months of symptoms and with a VAS score of more than 6 and plantar fascia thickness of 5 mm and failed conservative management. This is evidenced by a comparison of AOFAS, FAI score and thickness of plantar fascia using an ultrasonogram before and after the procedure. This study reflects better treatment outcomes with PRP injection compared to local steroid infiltration. This is the largest series of cases studied compared to other previously available studies in the literature. PRP injections may thus be used as a superior alternative to the already available treatments for chronic heel pain.

## Introduction

Plantar fasciitis causes pain which may be acute or chronic, in the inferior aspect of the heel at the attachment of the plantar fascia's medial band to the medial calcaneal tubercle [1]. Peak incidence of plantar fasciitis occurs between 40 and 60 years of age in both genders. Pain is sharp and gradual in onset along the medial aspect of the heel. It intensifies with the steps taken freshly out of bed in the morning while the pain abates as the person takes rest. Plantar fasciitis, previously considered an acute inflammatory disease, is now histologically understood to be a chronic degenerative process without inflammation [2]. The condition is diagnosed mainly based on clinical symptoms comprising of pain and tightness over the heel, so the diagnostic imaging is not routinely needed [3]. Rest, ice, stretching, orthoses, non-steroidal anti-inflammatory drugs, extracorporeal shock wave therapy, injections (of corticosteroids, botulinum toxin, dextrose, platelet-rich plasma) and surgery are commonly used in the management of the condition. Almost 90% of patients get better with non-surgical treatment [4]. Calcaneal osteophyte formation (heel spurs) on X-ray and thickened plantar fascia > 4.5 mm on magnetic resonance imaging or ultrasonogram are some of the typical imaging findings [3,5]. The non-surgical treatment which offers the best safety and efficacy in treating plantar fasciitis still remains undetermined. The release of plantar fascia surgically is not very common these days as its efficacy is variable [6]. In cases of resistant plantar fasciitis, corticosteroids are administered especially after the failure of conservative non-invasive interventions. They reduce the pain in patients with plantar fasciitis, effectively. Corticosteroid use furthermore may lead to rupture of the plantar fascia, skin depigmentation, fat pad atrophy, infections, peripheral nerve injury, muscle damage and post-injection flare. Platelet-rich plasma (PRP) encourages the healing process to happen naturally by the promotion of platelet growth factors and hastening the physiological healing. PRP is plasma enriched with platelets, which can stimulate bone and muscle healing. The tissue repair due to PRP is mediated by different types of cytokines and growth factors [4]. Clinically, PRP is widely used for healing in cardiac muscular injuries, neural injuries, tendinitis, plastic surgery and osteoarthritis [5]. There is a significant rise in the interest for the usage of growth factor containing plasma, for treating various inflammatory conditions. So PRP is administered as an alternative treatment for plantar fasciitis to reduce heel pain and to restore function. The purpose of this study is to assess the effective and safest treatment option for plantar fasciitis on the basis of changes in the outcomes by Visual Analog Scale (VAS) and subjective rating using the Roles and Maudsley score, functional outcome score by the Foot and Ankle outcome Instrument (FAI) core scale and American Orthopaedic and Ankle Society (AOFAS) ankle-hind foot scale. Ultrasonography is used for measuring the thickness of the plantar fascia based on two different treatments namely the corticosteroid (CS) and PRP injection. Plantar fasciitis along with comorbid conditions such as hypertension and diabetes was evaluated in this study. This study's findings could translate into a novel approach of handling chronic plantar fasciitis.

## Materials and methods

The present study was conducted between August 2018 and September 2020 in the Department of Orthopaedics, R. L. Jalappa Hospital and Research Centre, Tamaka, Kolar. Of the 110 patients with chronic plantar fasciitis, 55 patients were treated with corticosteroid (methylprednisolone) and the other 55 of them with PRP injection. Patients were considered for follow-up for a period of six months using VAS, FAI and Roles and Maudsley Score, AOFAS and ultrasonogram-based measurement of plantar fascia (PF) thickness.

Inclusion criteria

The following patients were accepted for the study: Patients who were diagnosed with more than three months’ duration of plantar fasciitis; patients who had a failure of the conservative management methods such as stretching exercises, non-steroidal anti-inflammatory drugs, and use of heel cushion pads for three months; patients who belonged to the Visual Analog Scale pain of values more than 6 (on a 10-point VAS) and the patients who had their plantar fascia thickness measuring >5 mm when assessed using ultrasonogram.

Exclusion criteria

The following patients were excluded from the study: Those patients who had a history of any previous surgeries in the ankle and foot; patients with a history of lower limb pathology/deformities including tarsal tunnel syndrome/ ankle effusion/Achilles tendinopathy/subtle cavus foot deformities, physiological flat foot deformities, and seronegative arthritis; pregnant patients and those with a history of recent aspirin intake or intake of any other similar non-steroidal anti-inflammatory drugs (NSAIDs).

Diagnosis of plantar fasciitis was made clinically according to the guidelines which include tenderness on palpation in the medial plantar or heel region. The pain usually begins while taking the first few steps after a period of inactivity and may worsen following weight-bearing for a prolonged duration and pain often gets increased by a recent weight gain that adds to the weight-bearing. The following investigations were done: plain radiograph - anteroposterior/oblique/lateral views, hemoglobin, bleeding time, clotting time, random blood sugar, serum urea, and serum creatinine.

**Figure 1:**
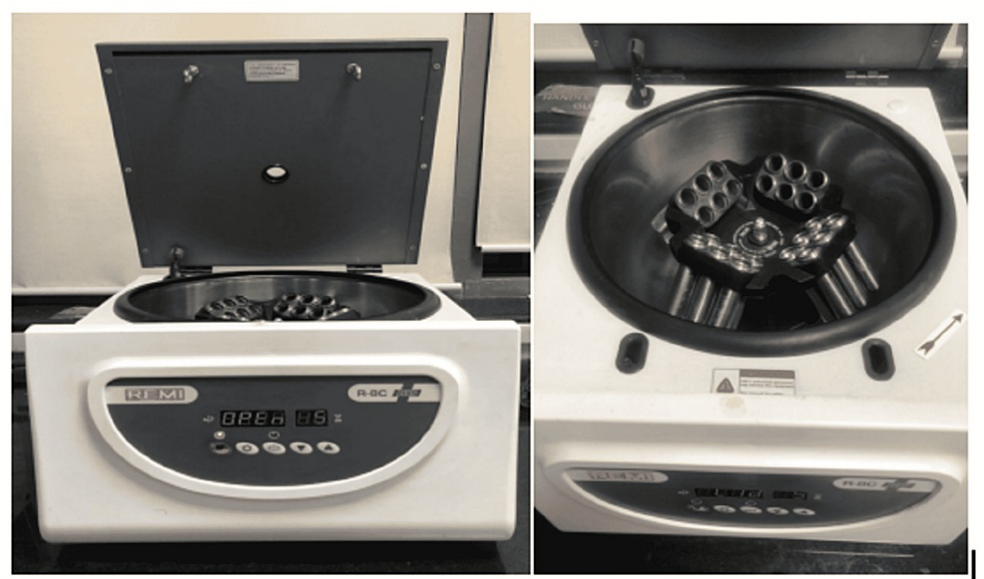
Laboratory centrifuge machine

**Figure 2:**
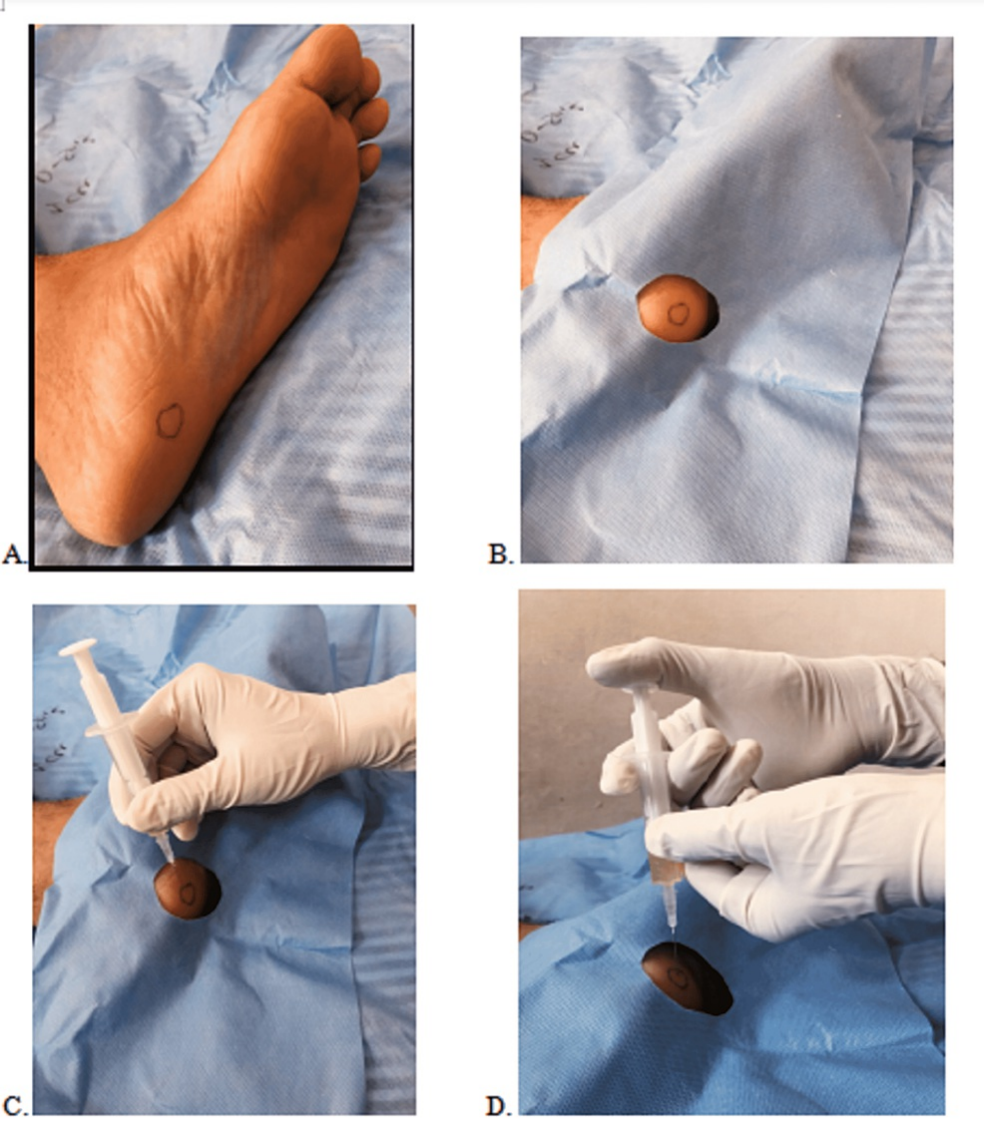
Platelet-rich plasma (PRP) injection procedure. A) Site of the injection at maximum point of tenderness. B) Injection part was painted and draped. C) Needle placed perpendicular to the site of the injection. D) PRP administered to cover the maximum area of tenderness.

Patients were randomly assigned into two groups. Baseline VAS/Foot Ankle score Instrument/American Orthopaedic and Ankle Society/Roles and Maudsley Score and ultrasonogram of the plantar fascia were evaluated. Odd-numbered patients were assigned to group A and these 55 patients received PRP injection. Even-numbered patients were in group B and these 55 patients received corticosteroid (methylprednisolone) injection. Aseptic precautionary measures were followed and using an 18-gauge needle, 27 ml of the patient’s peripheral whole blood was obtained [1]. A total of 3 ml of sodium citrate was added to the collected blood maintained in the ratio of 1:9. Using the double centrifugation technique at 1300 rpm for 10 minutes to separate erythrocytes and then again for 10 minutes at 3500 rpm to concentrate platelets by centrifugation, 3 ml of PRP was extracted. Figure 1 shows the Laboratory Centrifuge machine REMI R-8C Plus (REMI Sales & Engineering Ltd., Mumbai, India). PRP injections were given to group A. Corticosteroid - 2 ml (40 mg) methylprednisolone dissolved in 2 ml of sterile water - was injected in group B patients. PRP injection procedure is shown in Figure 2.

All injections (for PRP and corticosteroids) were given by the Orthopaedician, with the patient in the supine position and the ankle in the neutral position. The majority of patients received the injection in the medial plantar heel region and some patients received the injection at the maximum point of the tenderness.

Post injection protocol

**Figure 3:**
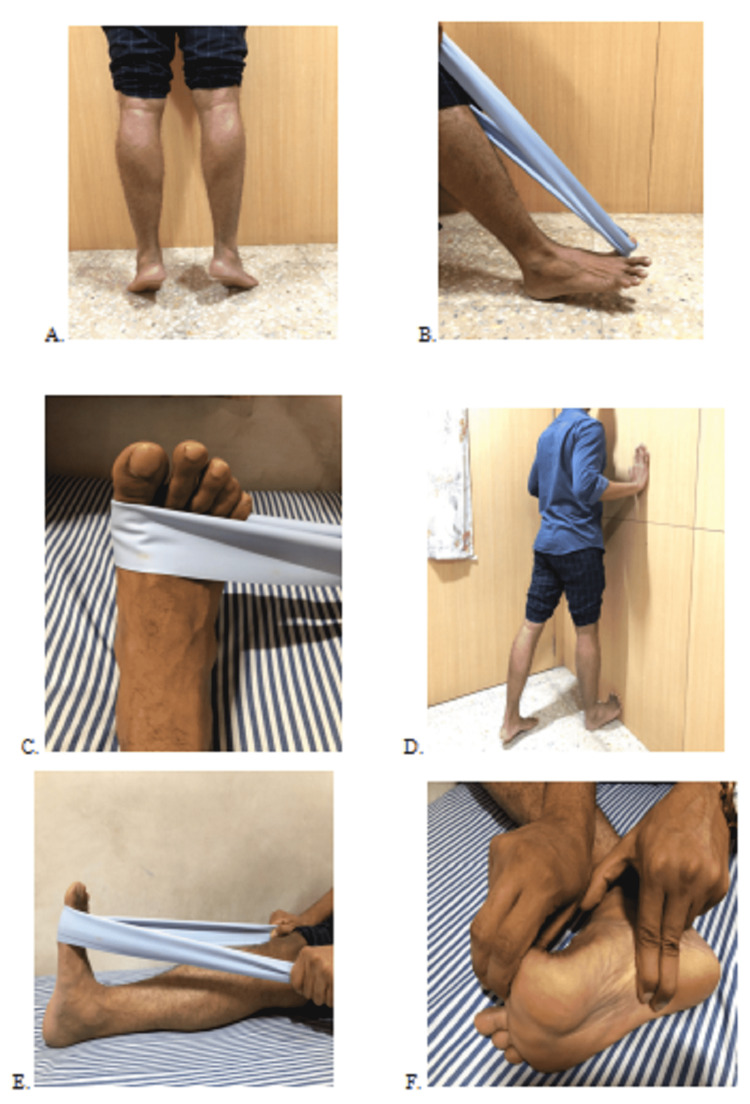
Plantar fascia - Strengthening & stretching exercise: (A) Rising of the heel. (B) Flexing the first toe against the elastic band. (C) Inversion of foot against the elastic band. (D) Using the toe balls, standing against a wall and stretching the calf muscles for 3 × 30 s. (E) While sitting on the heel with dorsiflexed ankle and toes for 3 × 30 s, stretching of the PF by kneeling. (F) For the next two weeks, manual stretching of the PF for 10 × 10 s

Patients on receiving the injection were to remain in a sitting position with the foot kept still for 15 minutes. Strengthening and stretching exercises were taught to patients. Patients were sent home with instructions to limit the usage of their feet for approximately 48 hours. The usage of non-steroidal medication was prohibited. After 48 hours, patients were advised to follow the standardized stretching protocol. Patients were instructed to perform the strengthening exercises slowly. The exercises included 3-4 seconds of concentric contractions followed by 3-4 seconds of eccentric contractions. They consisted of: (1) rising of the heel, (2) flexing the first toe against an elastic band, (3) inversion of the foot against an elastic band, (4) using the toe balls, standing against a wall and stretching the calf muscles for 3 × 30 s, (5) while sitting on the heel with dorsiflexed ankle and toes for 3 × 30 s, stretching of the PF by kneeling, (6) for the next two weeks, manual stretching of the PF for 10 × 10 s [7]. Following the stretching exercises, a formal strengthening program was initiated. Figure 3 shows plantar fascia strengthening and stretching exercises. Patients were allowed to proceed with normal sporting or recreational activities as much as they can tolerate, after four weeks of the procedure.

## Results

A total of 110 patients took part in this study. Screening and evaluation was done for 110 painful heels and the patients were followed up (There were 10 drop-outs from the study, five from each group). With each follow-up, clinical, subjective, radiological and functional outcomes were being assessed at the first month, third month, and sixth month by using VAS/FAI and Roles and Maudsley Score, AOFAS and ultrasonogram plantar fascia. In our study, 59 were females and 41 were males. A total of one, 63, 36 and 0 patients had BMI in the ranges of <18.5, 18.5-24.9, 25-29.9 and 30-34.9, respectively. Most of the patients were in the normal weight range of 18.5-24.9, with their mean BMI being 23.6. Two patients had post-operative complications (superficial infection) in the PRP injection group, while 10 patients had post-operative complications (five patients developed superficial infections, three patients developed skin depigmentation, and two patients had atrophy of fat pad) in the corticosteroid injections (CSI) group. Infection subsided for patients in both the groups on subsequent follow-up.

The mean age of groups of patients who were given platelet-rich plasma and CSIs was 46.74 ± 12.45 years and 48.5 ± 10.39 years, respectively. VAS score prior to an injection showed a p-value of 0.0486 and the VAS score post-injection at the first, third and sixth month showed a p-value <0.001. The mean VAS values before the injection, at the first month, third month and sixth month in the PRP group, were 7.32 ± 0.587, 5.78 ± 0.679, 4.52 ± 0.505, 3.5 ± 0.614, respectively. The mean VAS values before the injection, at the first month, third month and sixth month in the corticosteroid (CS) group, were 7.24 ± 0.555, 6.46 ± 0.813, 5.64 ± 0.693, 4.44 ± 0.501, respectively. Final outcomes of the mean difference between PRP and CS for continuous variables are shown in Table 1. Hence, significant betterment was seen in patients belonging to the PRP injection group. In the assessment of the PRP group with subjective ratings using Roles and Maudsley score, at the first month follow-up, the result was excellent in 10 patients and good in 16 patients, and in the corticosteroid group the result was excellent in 17 patients and good in 17 patients. At third month follow-up in the PRP group, the score was excellent in 17 patients and good in 21 patients, and in the corticosteroid group, the score was excellent in 0 patient and good in 11 patients. At the sixth month follow-up in the PRP group, the score was excellent in 32 patients and good in 13 patients, and in the corticosteroid group excellent in six patients and good in two patients (rest of the patients had fair and poor outcomes according to the Roles and Maudsley score). While assessing functional outcomes using AOFAS and FAI Score, the mean AOFAS of the subjects who were given injections of PRP (pre-injection, first month, third month and sixth month) were 59.58, 70.74, 82.20, 92.04 respectively, and the mean score of AOFAS of the subjects in CSIs (pre-injection, first month, third month and sixth month) was 56.62, 64.08, 71.22, 76.08, respectively. p-value is statistically significant between the groups (AOFAS at first month, third month and sixth month) as the p-value is <0.001.

**Table 1:** Final outcomes of mean difference between PRP and CS for continuous variables PRP - Platelet-rich plasma; CS - Corticosteroid; SD - Standard deviation; VAS - Visual Analog Scale; AOFAS - American Orthopaedic Foot and Ankle Society; FAI - Foot and Ankle Outcome Instrument Core Scale.

	PRP		CS		p-value
	MEAN	SD	MEAN	SD	
VAS SCORE AT 6 MONTHS FOLLOW-UP	3.50	0.614	4.44	0.501	0.001
AOFAS SCORE AT 6 MONTHS FOLLOW-UP	92.04	3.86	76.08	5.05	0.001
FAI SCORE AT 6 MONTHS FOLLOW-UP	41.10	5.34	68.0	4.00	0.001
PLANTAR FASCIA THICKNESS AT 6 MONTHS FOLLOW-UP	3.24	0.431	5.06	0.512	0.001

The mean FAI scores of the subjects in the PRP injections group (pre-injection, first month, third month and sixth month) were 100.58, 81.54, 61.86, and 41.10 respectively, and the mean FAI scores of the subjects in the CSIs group (pre-injection, first month, third month and sixth month) were 100.14, 80.84, 73.40, and 68.00 respectively. FAI score was statistically significant between groups in the third month and sixth month as p-value <0.001. On assessing radiological outcomes using plantar fascia thickness measurement, the mean thickness of plantar fascia of the patients who were given PRP injections (pre-injection, first month, third month and sixth month) was 6.02, 4.96, 4.06, and 3.24 respectively and the mean thickness of plantar fascia of the subjects in CSIs group (pre-injection, first month, third month and sixth month) was 6.30, 5.28, 5.12, 5.06 respectively. p-value is statistically significant between the groups in measuring plantar fascia thickness at the first month, third month and sixth month as the p-value is <0.001.

## Discussion

Plantar fasciitis is a regular musculoskeletal disorder faced during the orthopaedic day-to-day practice. Plantar fasciitis with heel pain, be it acute or chronic, is quite debilitating. It considerably affects the quality of life of patients. There have been quite a few treatment methods in practice. Physiotherapy and bracing are usually recommended. CSIs whose efficacy is still conflicting lead to local and permanent damage to the fascia and are still being used extensively. With the arrival of biological treatments into orthopaedics, PRP has been used in many clinical situations including bone graft augmentation, healing of wounds, wound hemostasis, boosted healing of anterior cruciate ligament injuries and augmentation of treatment of tendinosis. In persistent cases of plantar fasciitis, weight loss should be recommended [8]. A research concluded that persons with plantar fasciitis have thicker plantar fascias than people without heel discomfort. In plantar fasciitis, the appearance of the plantar fascia on ultrasonic examination revealed inflammatory alterations [9] while another research adds to our understanding of functional stability in the medial column of the foot [10].

**Figure 4:**
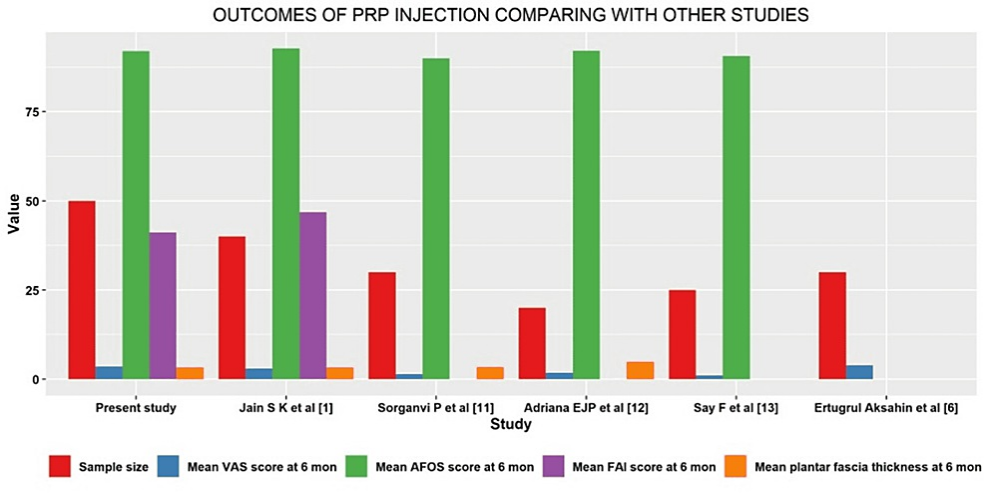
Bar diagram showing outcomes of PRP injection compared with other studies mon - months; PRP - Platelet-rich plasma; VAS - Visual Analog Scale; AOFAS - American Orthopaedic Foot and Ankle Society; FAI - Foot and Ankle Outcome Instrument Core Scale

**Figure 5:**
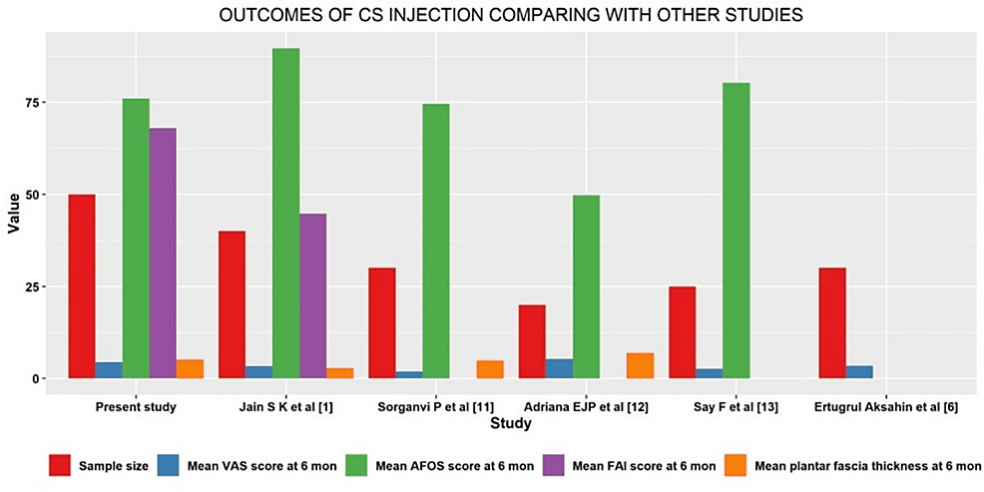
Bar diagram showing outcomes of CS injection compared with other studies mon - months; CS - Corticosteroid; VAS - Visual Analog Scale; AOFAS - American Orthopaedic Foot and Ankle Society; FAI - Foot and Ankle Outcome Instrument core scale.

In comparison with other studies, our study's sample size was higher and we have used four parameters to assess the disease as accurately as possible by clinical, subjective, radiological and functional outcomes at the first month, third month, and sixth month by the usage of VAS/FAI, Roles and Maudsley Score, AOFAS and ultrasonogram of the plantar fascia. Two other Indian studies have been compared. Jain et al. found that PRP and corticosteroid were equally effective [1]. In his study, he used all four parameters. Soraganvi et al. used only AOFAS functional score, in which the functional and clinical outcomes showed better results in the PRP group [11]. Assessing plantar fascia thickness radiologically showed almost similar outcomes in both PRP and CSI groups. Among American and European studies, Jiménez-Pérez et al. compared both PRP and CS injection in PF. But they used only 20 patients in either of the groups with only one functional outcome scale. PF thickness in the PRP group was >4.82 mm at the six-month follow-up, a higher value than the normal cut-off of >4 mm [12]. Among the Asian studies, Say et al. used only 25 patients in each group with only VAS and AOFAS assessment, without radiological assessment of PF thickness [13]. Akşahin et al. used 30 patients in both the groups, measuring only VAS (Table 2) [6]. Bar diagrams show outcomes of PRP and CS injections in comparison with other studies in Figure 4 and Figure 5, respectively. Surgical management is advised only when all the conservative therapies fail. Only 5 to 10% of cases progress to surgery [14]. The effectiveness of orthoses in the treatment of plantar fasciitis is still inconclusive [15].

**Table 2:** Comparison with other studies “-” parameter not mentioned in the study. PRP - Platelet-rich plasma; CS - Corticosteroid

	Present Study		Jain et al. [1]		Soraganvi et al. [11]		Jiménez-Pérez et al. [12]		Say et al. [13]		Akşahin et al. [6]	
	PRP	CS	PRP	CS	PRP	CS	PRP	CS	PRP	CS	PRP	CS
SAMPLE SIZE	50	50	40	40	30	30	20	20	25	25	30	30
MEAN VAS SCORE AFTER INJECTION AT 6 MONTHS	3.5	4.4	3.0	3.3	1.4	1.9	1.8	5.3	1.0	2.6	3.9	3.4
MEAN AOFAS SCORE AFTER INJECTION AT 6 MONTHS	92	76	92.7	89.6	90	74.6	92.1	49.7	90.6	80.3	-	-
MEAN FAI SCORE AFTER INJECTION AT 6 MONTHS	41.1	68	46.8	44.7	-	-	-	-	-	-	-	-
MEAN PLANTAR FASCIA THICKNESS AT 6 MONTHS	3.2	5.06	3.2	2.8	3.3	3.7	4.82	6.90	-	-	-	

**Figure 6:**
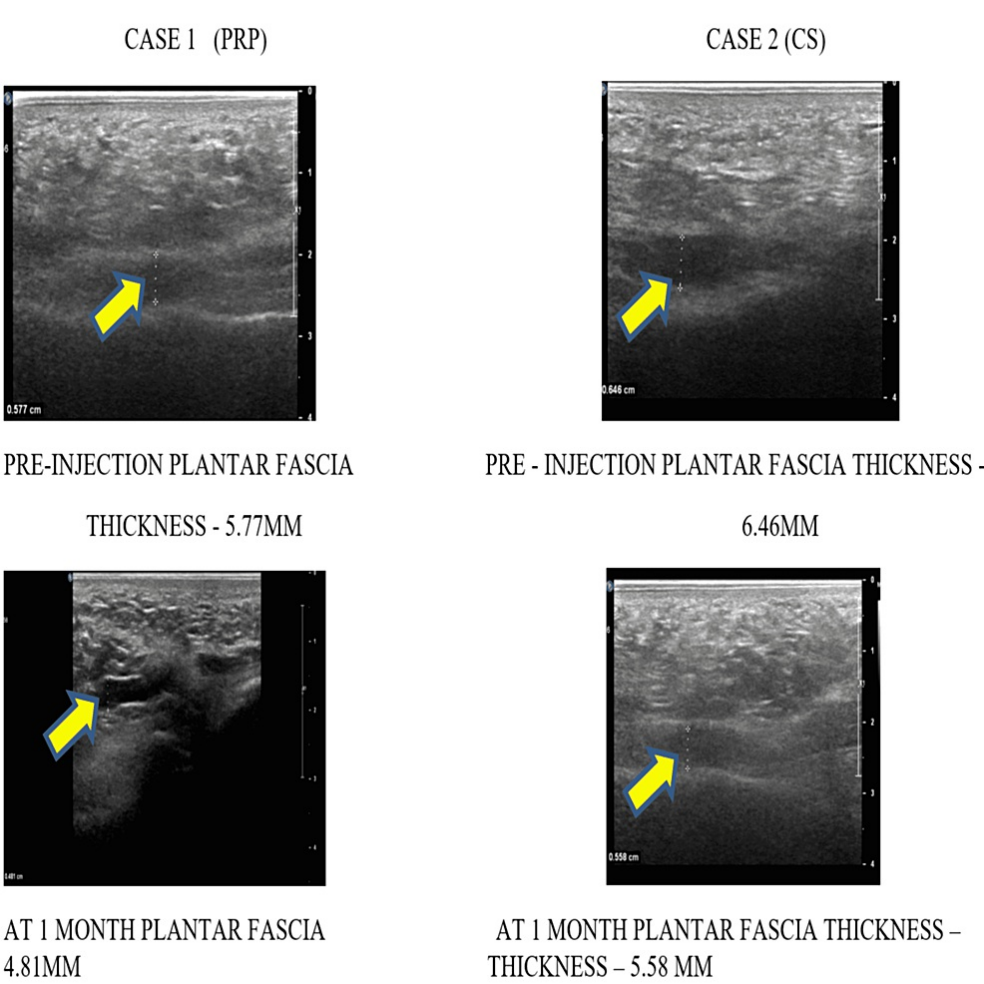
Ultrasonic measurement of plantar fascia thickness during pre-injection and at one month in PRP vs CS groups Yellow arrow mark showing plantar fascia thickness measurement.

**Figure 7:**
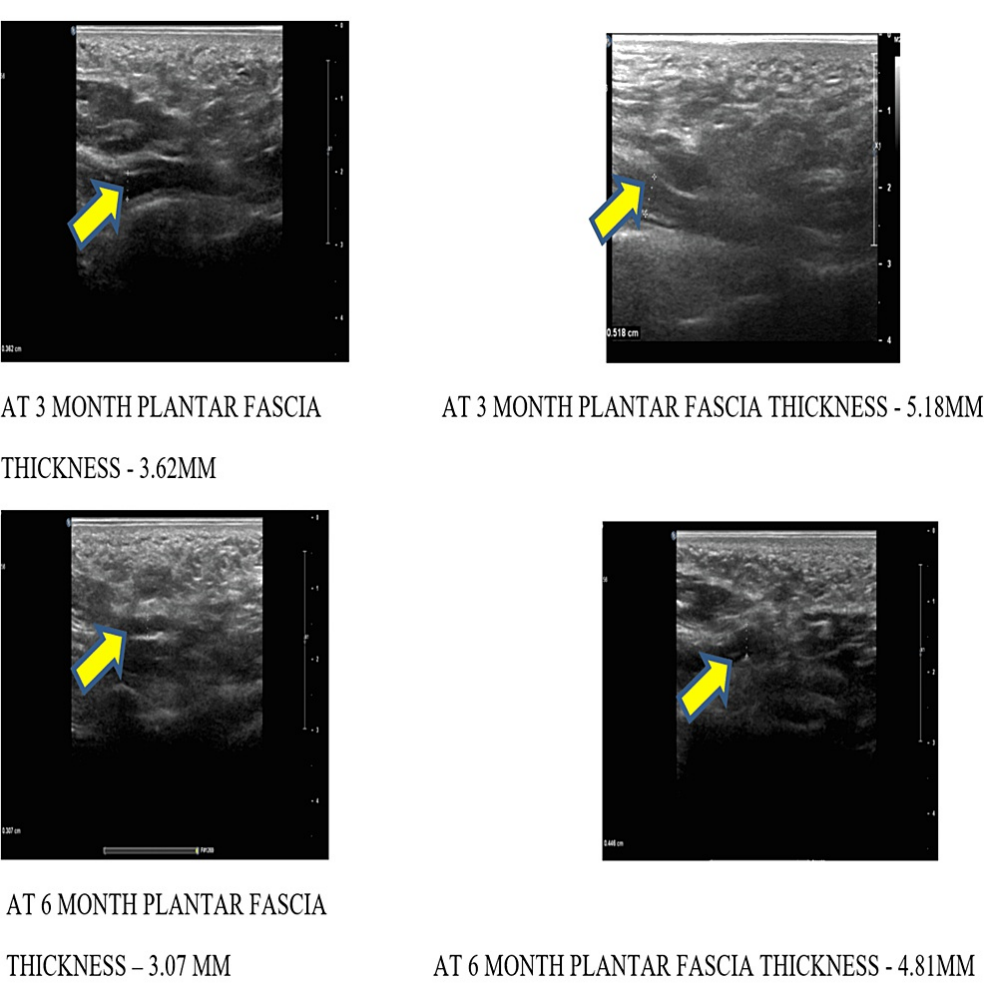
Ultrasonic measurement of plantar fascia thickness during third and sixth month in PRP vs CS groups Yellow arrow mark showing plantar fascia thickness measurement.

In our study, significant improvement was seen in the PRP injection group when compared with the CSI group, although steroid injections show significant improvement in clinical, subjective rating, functional and radiological outcomes one month after injection. However, for long-term effects, PRP injection gives better results in clinical, subjective rating, functional and radiological outcomes during six months when compared to the corticosteroid group. Post-operative complications were minimal in the PRP group in comparison with the corticosteroid group. Ultrasonic measurements of plantar fascia thickness in PRP vs CS groups are shown in Figure 6 and Figure 7.

Based on the analysis of our study, we suggest the following treatment plan, based on the degree of disability as assessed by the scoring below, could be considered (Table 3).

**Table 3:** Treatment plan for plantar fasciitis

Class	Description	Clinical Rating (VAS) Scale	Radiological Assessment (Plantar Fascia Thickness)	Treatment
I	>3-month symptom duration + some discomfort following activity	1-5	4 to 5 mm	Observation
II	>3-month symptom duration + failed conservative + some discomfort following activity	6-8	>5.1 to 7 mm	PRP Injections + Stretching and strengthening exercise of the plantar fascia

## Conclusions

Our study findings prove that PRP is a good method of managing patients with chronic plantar fasciitis, presenting with some discomfort following activities, with more than three months symptom duration, with a VAS score of more than 6 out of 10, plantar fascia thickness of 5 mm and failed conservative management. This is evidenced by a comparison of AOFAS, FAI score and ultrasonogram of plantar fascia thickness before and after the procedure. This study has shown better results with PRP injection compared to the local steroid infiltration. This is the largest case series studied, compared to the previously available studies in the literature. PRP injection may be thus used as a better alternative to the treatments available currently for chronic heel pain.

This research was constrained by brief follow-up of patients, lack of a control group and a restricted patient population. In order to provide a clearer insight into the effectiveness of both treatment types, a randomized controlled trial with a larger population, a longer follow-up, and a control group would be needed.
